# Honey: Inorganic Composition as Possible Marker for Botanical and Geological Assignment

**DOI:** 10.3390/molecules30071466

**Published:** 2025-03-26

**Authors:** Paolo Inaudi, Matteo Garzino, Ornella Abollino, Mery Malandrino, Agnese Giacomino

**Affiliations:** 1 Department of Drug Science and Technology, University of Torino, Via Pietro Giuria 9, 10125 Turin, Italyagnese.giacomino@unito.it (A.G.); 2 Department of Chemistry, University of Torino, Via Pietro Giuria 7, 10125 Torino, Italy; mery.malandrino@unito.it

**Keywords:** honey, geographical origin, botanical origin, metal content, chemometric

## Abstract

Honey can be classified based on its geographical or botanical origin according to Directive 2001/110/EC. Geographical origin is determined when the pollen collection occurs entirely in a specific location, allowing producers to label the honey with the name of the region. Verification typically involves melissopalynological analysis to match the pollen spectrum with regional vegetation. Botanical origin applies when honey predominantly comes from a single floral species, characterized by specific organoleptic, physico-chemical, and microscopic properties. However, defining “predominantly” and identifying distinct physico-chemical parameters remain ambiguous. This review evaluates the use of chemical analysis as a complement or alternative to melissopalynological methods for determining honey’s origin. The focus is on inorganic composition, particularly metals and semimetals, as potential fingerprints to identify botanical or geographical provenance. Relevant studies were reviewed, with data reprocessed and analyzed using chemometric techniques, including Principal Component Analysis and Agglomerative Cluster Analysis. These methods reveal common traits distinguishing honeys by origin. Chemical analysis combined with chemometric processing enhances honey identification, prevents fraud, assesses environmental pollution in collection areas, and evaluates the impact of processing on the final product.

## 1. Introduction

The control system currently used to verify the veracity of geographical denomination is melissopalynological analysis, which allows us to identify whether the pollen spectrum found is consistent with the vegetation [1,2]. The botanical designation applies when honey is predominantly derived from a single floral species and exhibits corresponding organoleptic, physico-chemical, and microscopic characteristics [3]. The problem, in this case, is that the physico-chemical parameters able to discriminate between honey from one floral species and honey from another one are insufficiently documented in the literature, just as it has never been clarified what was meant by “mainly” (e.g., “mainly chestnut”), leaving this term open to “free interpretation”. The analysis of the geographical and botanical origin of honey, as well as other food products, plays a critical role in ensuring authenticity and safeguarding against adulteration. This process is fundamental for maintaining quality standards, protecting consumer trust, and supporting regulatory compliance within the food industry [4,5,6]. The aim of this review is to evaluate the potential of analytical chemical approaches in identifying the botanical species or geographical origin of honey. This is proposed as a valid alternative or complementary method to melissopalynological analysis, which is traditionally used for such purposes. Specifically, the focus was placed on the inorganic composition of honey to investigate whether the content of inorganic elements could serve as a reliable fingerprint for identifying the origins of different honeys. To achieve this objective, an extensive bibliographic search was conducted to identify relevant studies in the field. The search was performed using scientific databases, namely Scopus and Web of Science, with the use of defined keywords such as “honey”, “inorganic composition”, “geographical origin”, “botanical origin”, “metals”, and “semimetals”. Articles were included based on specific criteria: (i) they provided quantitative data on inorganic element concentrations in honey; (ii) they addressed either the geographical or botanical provenance of honey; and (iii) they were published in peer-reviewed journals within the last 15 years, to ensure up-to-date and relevant findings. Studies lacking sufficient data or not meeting the inclusion criteria were excluded. The selected literature was divided into two main categories: studies focusing on the geographical origin of honey and those examining botanical provenance. For each study, relevant data on the concentrations of metals and semimetals were extracted and systematically compiled. To ensure consistency, the reported values for the same type of honey or honeys from the same country were averaged, creating a unified dataset for further analysis. The collected data were processed using chemometric techniques. Principal Component Analysis (PCA) was employed to highlight the most significant variables contributing to the differentiation of honeys based on their inorganic profiles. Additionally, Agglomerative Cluster Analysis (ACA) was applied to group samples and detect similarities within the dataset, aiding in the identification of common features that could distinguish honeys according to their geographical or botanical origins. This systematic approach provides a comprehensive overview of the inorganic profiles of honey, integrating data from a wide range of studies.

### 1.1. Honey

Honey is a complex substance produced by bees from plant-based materials such as nectar or honeydew [7]. According to Annex I of the European Council Directive 2001/110 [8], honey is defined as a natural sweet product produced by honeybees (*Apis mellifera*) from the nectar of plants or from the secretions of plant-sucking insects found on living parts of plants. The bees collect these substances, combine them with specific enzymes, transform them, dehydrate, store, and allow them to mature in the honeycombs of their hives. The nectar, found in flowers, serves to attract pollinators and facilitates the fertilization of the plant. Bees can only collect nectar from specific flowers, known as melliferous plants, whose floral structure—specifically the ligule—is of an appropriate size for them to access. The nectar is accumulated into a specialized portion of the bee’s digestive system, called the crop (or honey stomach), which serves as a temporary storage site for collected food. Within the crop (ingluvium), nectar serves as a substrate for invertase enzymes, initiating the process of honey production by hydrolyzing sucrose into glucose and fructose. Upon returning to the hive, the bee regurgitates the nectar, and a process known as trophallaxis occurs. This involves the communal sharing of food within the bee colony, facilitating an increase in enzyme concentration and a decrease in nectar moisture content. The final stage in honey production involves dehydration, achieved through the deposition of nectar in thin layers along the hive walls and aided by “fanning” bees, which create airflow to further enhance moisture evaporation. Honey is considered mature after approximately thirty-six days, although this timeframe can vary depending on environmental factors such as humidity, temperature, and the initial moisture content of the nectar. The composition of honey, particularly the ratio of nectar to honeydew, is influenced by various factors including vegetation type, flowering period, foraging insect species, and the timing of honey collection by beekeepers [9]. Honey is broadly categorized into two types: monofloral and multifloral (or wildflower). Monofloral honey is derived primarily from a single botanical species, typically when the plant is present in abundance in the foraging area [10]. Producing this type of honey is more challenging and demands significant effort from beekeepers, who must relocate the bee colonies to specific fields just before the anthesis period. Additionally, honey must be processed immediately after the end of the flowering period of the selected species. However, honey is classified as multifloral when it is derived from various botanical species and represents a blend of different nectars.

### 1.2. Adulteration and Directive

Every year, approximately 1.83 million metric tons of natural honey are produced worldwide. The European Union (EU) is the second largest producer of honey in the world after China, with the majority of Europeans consuming honey on a weekly basis. Despite having approximately 19 million beehives within the EU, the region meets only 60% of its honey consumption needs [11,12,13]. This means that the EU relies on the importation of honey from countries outside of the EU as well as on the movement of large quantities of honey between Member States. Honey within the EU market must adhere to the quality and labelling rules outlined in the updated “Breakfast Directives” that define the standards and composition criteria of this food. The revision of the Honey Directive incorporates the mandatory origin labelling for honey. It enables the Commission to “introduce harmonised methods of analysis to detect honey adulteration with sugar” in collaboration with the Commission’s Joint Research Centre (JRC), to regulate composition criteria of honey, prohibiting the removal of pollen and the overheating of honey, and means that the Commission can put in place traceability methods to ensure that geographical origin information is passed on correctly from the beekeeper all the way through to the consumer [14,15,16,17,18]. Honey is often falsified using glucose syrups derived from beetroot or corn, palm sugar, invert sugars, or syrups with high concentrations of fructose [19]. Adulteration of honey can occur directly, by adding honey sugars at increasing concentrations (7%, 15%, and 30%) to pure honey, or indirectly, by feeding the bees with these sugars; in this way adulteration occurs through the physiological digestive process of the bee; a third method of fraud is to add a lower quality honey to a fine one, such as the addition of rapeseed honey to that of acacia [20]. Other frauds can concern the starting botanical species or the territoriality of the honey.

### 1.3. Analytical Techniques Commonly Used for Honey Origin Determination

The assessment of the origin of a honey sample is based on the determination of components and/or properties considered most characterizing [21]: (i) chemical–physical characterization (pH, Optical Rotation [22], sugars composition [23], enzymes, colour [24]); (ii) mineral content [25]; (iii) carbohydrate composition [26], amino acids [27], phenolic compounds [28], and other components [29]. The most adopted techniques are (i) Fourier Transform—Near InfraRed (FT-NIR) spectroscopy on non-extracted samples [30]; (ii) Fourier Transform InfraRed attenuated total reflectance (FTIR-ATR) spectrometer equipped with a deuterated triglycine sulfate (DTGS) detector on non-extracted samples [31]; (iii) emission spectroscopy [32]; and (iv) Nuclear Magnetic Resonance (NMR) spectroscopy on extracted samples [33,34]. In particular, the following methods can be used to determine the botanical origin of honey: (i) high-performance liquid chromatography (HPLC) determination of phenolic compounds and flavonoids [28,35]; (ii) HPLC determination of amino acids [36]; and (iii) Gas Chromatography-Mass Spectrometry (GC-MS) determination of volatile [37] and phenolic compounds [38]. The use of statistical data processing consists in selecting groups of homogeneous samples from which a standard profile will emerge for every type studied, each with its own organoleptic, microscopic and chemical–physical characteristics. So, it is possible to use some characterization sheets of the main single-flower honeys based on microscopic, organoleptic, and chemical–physical analyses. Control of botanical origin is therefore based on the comparison between the data obtained on the sample to be evaluated and the profiles already identified: if there is an overlap, the honey can be defined as unifloral. The assessment of the geographical origin is more difficult due to small differences in composition and not all the above methods can be effective.

### 1.4. Chemical Composition

**Figure 1:**
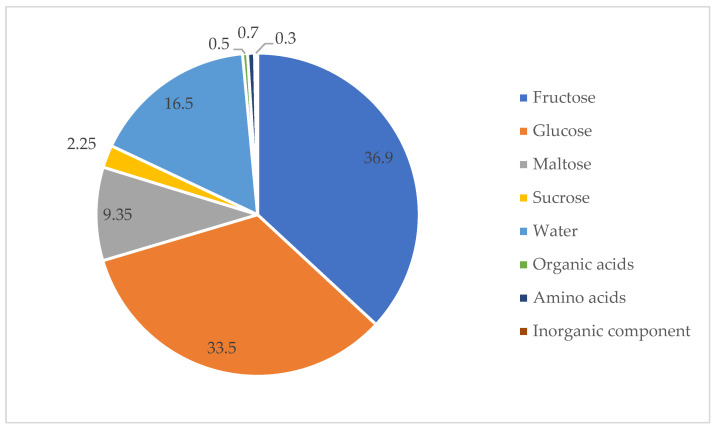
Honey composition. (All figures presented in this work were created by the authors based on the data analyzed).

The chemical composition of honey (Figure 1) varies depending on its botanical origin and the chemical properties of the nectar from which it is derived. However, it primarily consists of a mixture of carbohydrates, including fructose (27.3–44.3%), glucose (22.0–40.8%), maltose (2.7–16.0%), and sucrose (1.5–3.0%) [39].

The ratio of glucose to fructose can serve as a classification parameter for different unifloral honeys, whereas sugars present in smaller amounts are less useful for identification, as they are primarily produced through enzymatic reactions, notably by invertases [40]. Additionally, honey contains over 25 different oligosaccharides (tri- and tetrasaccharides), which, while not influencing its organoleptic properties, can be employed as markers for botanical identification [41]. For instance, quercitol is characteristic of the genus *Quercus* spp., while perseitol (heptane-1,2,3,4,5,6,7-heptol that has R-configuration at positions 2, 3, 4 and 5, and S-configuration at position [42]) is a marker of avocado honey. The formation of di- and monosaccharides in honey occurs both due to the action of the microbiota and enzymes present in the intestinal tract of bees, as well as during the maturation process within the hive. The primary enzyme in the bee’s intestine is invertase (sucrase), which hydrolyzes sucrose into glucose and fructose. This reaction leads to a shift in optical rotation from right-handed to left-handed, hence the enzyme’s name, invertase. Other enzymes found in honey include glucose oxidase, which, in the presence of oxygen, catalyzes the conversion of glucose into gluconic acid and hydrogen peroxide:

β-D-glucose + O_2_ ⇄ D-glucono-1,5-lactone + H_2_O_2_

Then catalase breaks down hydrogen peroxide into water and oxygen:

2 H_2_O_2_ ⇄ O_2_ + 2 H_2_O

Finally, diastases catalyze the hydrolysis of oligosaccharides into simple sugars [43]. Enzymes serve as crucial markers for assessing the freshness of honey since they degrade over time and with heat treatment. Water concentration in honey depends on the initial botanical source and the degree of maturation in the hive. In monofloral honey, the water content ranges from 16.0% to 17.5%, while in multifloral honey it averages around 17%. The maximum allowable moisture content set by Codex Alimentarius is 20% [44]. Water presence influences crystallization, viscosity, and shelf life by potentially promoting fermentation. Honey also contains small amounts of organic acids, ranging from 0.1% to 1%. The principal organic acid is gluconic acid, which, as previously mentioned, is formed through the oxidation of glucose-by-glucose oxidase in the presence of oxygen. Other organic acids found in lower concentrations include malic, succinic, formic, maleic, acetic, and butyric acids. Most honey is acidic, with a pH typically between 3.3 and 4.6 for flower-derived honey, except for chestnut honey, which has a pH between 5 and 6. Honeydew honey has a higher pH, between 4.5 and 6.5, due to its higher mineral content. Amino acid and protein content in honey is low, reaching a maximum of 0.7%. The main amino acid is proline, which is used as an indicator of honey maturity. Other amino acids present include phenylalanine, tyrosine, glutamic acid, asparagine, and aspartic acid [45]. The protein fraction is primarily composed of enzymes.

The volatile and semi-volatile fractions of honey play a crucial role in its classification. These compounds, including aldehydes, alcohols, esters, and phenols, are low molecular weight molecules that define honey’s aromatic profile. For example, 2-(5-ethenyl-5-methyloxolan-2-yl)-propanal is a marker for *Citrus* spp. honey, and p-anisaldehyde is characteristic of chestnut honey. Although studying the volatile profile allows us to distinguish honeys of different geographical origins, several factors can interfere with an accurate classification. The origin of honey, beekeeping practices, and environmental conditions can alter the aromatic composition. Additionally, even monofloral honeys may contain volatile elements from other botanical species [46]. Environmental pollutants, such as pesticides and polycyclic aromatic hydrocarbons (PAH), can also be present in the volatile fraction, including 1,2-dibromoethane, 1,4-dibromobenzene, and naphthalene [47]. Hydroxymethylfurfural (HMF) is not naturally present in fresh honey; it is a degradation product of fructose. HMF is an important indicator of freshness and proper storage conditions. The legal limit for HMF content, as per Legislative Decree 179/04, is 40 mg/kg [48].

## 2. Chemical–Physical Properties

The chemical and physical properties of honey are influenced by its composition and, therefore, by its botanical origin. The composition can be evaluated through the combination of different physical and chemical parameters with multidimensional data analysis [43]. In recent years, to safeguard the high quality of honey from adulteration, specific regulations from the European Union with Directive 2001 have been included [8].

### 2.1. Free Acidity and pH

Honey has an acidic pH that oscillates between 3.5 and 5.5, due to the presence of inorganic ions and organic acids which also contribute to its stability against bacterial agents. The acid contained in higher concentrations is gluconic acid, which is present in equilibrium with its lactone form. The evaluation of free acidity is obtained by titration of the sample with 0.1 M sodium hydroxide and must not exceed 50 mEq/kg [49].

### 2.2. Optical Activity

Certain sugars can rotate the plane of polarized light; in particular, fructose rotates it in a negative direction [α] = −92.3 and glucose in a positive direction [α] = +52.5. The final rotation of the sample depends on the ratio of the two sugars. The evaluation of optical activity allows us to easily distinguish honeydews, which are dextrorotatory, from nectar honeys, which are levorotatory [50].

### 2.3. Color

The color of honey varies from white or pale yellow to amber or black. It depends on many substances present in the matrix, including carotenoids, pigments such as chlorophyll, and phenolic compounds [43]. The color is also affected by metal content; indeed, dark-colored honeys would have a higher concentration of metals compared to lighter ones. Dark colors (chestnut and honeydew honey) are associated with high concentrations of metals such as Cd, Fe and Pb, while light ones (eucalyptus and thyme) are associated with Al and Mg [51].

The antioxidant activity of honey is also associated with color; in fact, the increase in color intensity is associated with greater antioxidant power [52]. The color of honey is an important parameter for the identification of unifloral honeys, as it is linked to the botanical species. Alterations in color may be due to incorrect practices of the beekeeper, exposure to light during storage, and the aging of the honey itself. The color of honey is evaluated using a colorimeter and is expressed on the Pfund scale. Light colors reach 20 mm on the Pfund scale, while dark honeys reach values over 100 mm [53,54].

### 2.4. Ash Content

The ash content is obtained by burning the honey sample in special stoves at a temperature of 550 °C. The ash is mainly composed of the inorganic materials present in the sample, such as, for example, the mineral component. The result is expressed in g/100 g of sample [55]. The ash content should not exceed 0.6% for common honeys, and not more than 1.0% for honeydew honey or mixtures of honeydew honey and blossom honey [56].

### 2.5. Electrical Conductivity

Electrical conductivity depends on the concentration of ionizable materials present in the sample, therefore, in honey; on the presence of minerals; and to a small extent also on proteins and dissociated acids. Over time, this analysis replaced the ash content. The electrical conductivity in honey must not exceed 0.8 mS/cm, while in honeydew and chestnut honey it must have a higher value. This is due to the nature of honeydew, since it is formed by insects that directly suck the phloem of the plant, which is richer in minerals than flower nectar [57].

### 2.6. Density

Density is defined as the ratio of mass to volume of a given substance. In honey, this parameter varies between 1.38 and 1.45 g/cm^3^ at 20 °C [58].

### 2.7. Viscosity

Honey is a viscous liquid, and this property is influenced by the water content present and the temperature. As the concentration of water increases, the viscosity decreases. The high viscosity and wettability of honey are the causes of its being sticky [59].

### 2.8. Melting Point

The melting point of crystallized honey is between 40 °C and 50 °C. Since honey only exists below its melting point, it is defined as a supercooled liquid and is characterized by not freezing at low temperatures. These values, however, fluctuate depending on the composition of the honey itself. Below the melting point, honey is in a condition of metastability, or in an energy minimum, which will last until a crystalline nucleus is provided to the system, or in a supersaturated state of sugars, in which it crystallizes spontaneously [60]. Crystal nuclei tend to form more quickly when the honey is subject to mechanical phenomena such as stretching, agitation or mixing; even honeys characterized by a high glucose/fructose ratio tend to crystallize faster, since glucose tends to aggregate. The optimal crystallization temperature is approximately 14 °C, as it is almost inhibited below 5 °C and above 25 °C. Below 5 °C, the viscosity is so high that it prevents the precipitation and nucleation of glucose crystals, while above 25 °C there is partial destruction of the crystals.

### 2.9. Humidity

Hygroscopy is the ability of a substance to absorb water. The fructose present inside honey is highly hygroscopic and this allows it to maintain a balance with humidity present in the air; in fact, the quantity of water present depends on the environment. Also, there must not be too much humidity, to avoid fermentation processes by naturally present yeasts. Fermentation starts more easily when the percentage of water is higher than 25% and in crystallized honey, where the glucose concentration decreases and promotes an environment suitable for the development of yeasts [61].

### 2.10. The Inorganic Component

The inorganic composition of honey is influenced by the environment in which the plants harvested by bees grow, in particular by the composition of three compartments: soil, water, and air. The Earth’s crust, made mostly of oxides like silicon, aluminum, calcium, potassium, iron, and sodium, is divided into continental and oceanic crust. The continental crust is thicker (35 km on average, up to 90 km in mountains) than the oceanic crust (6 km thick). Water, essential for life, exists in various forms: meteoric (rain, snow), surface (rivers, lakes), and underground (aquifers, springs). Meteoric water is the purest but can become polluted as it passes through the atmosphere. Underground water, enriched with minerals, becomes spring water when it resurfaces. Water contains suspended materials, colloids, and dissolved species, with pollutants from industry, agriculture, and pesticides, like phosphates, which can cause harmful algae blooms.

The atmosphere has evolved over billions of years, now primarily composed of oxygen, nitrogen, and argon, with additional pollutants from human activities. These pollutants, such as carbon monoxide (CO) and high concentrations of carbon dioxide (CO_2_), can negatively affect human health [62].

In the context of honey, it is also important to mention heavy metals, such as cadmium, mercury, chromium, and lead, which can be extremely toxic even at very low concentrations [63]. Mercury, for example, is primarily produced by volcanic eruptions, augmented by industrial waste; it passes through the rainwater from the atmosphere to aquifers and soil. Mercury poisoning mainly occurs through the ingestion of contaminated food, especially fish. Administration of small and chronic doses can cause acrodynia, a condition characterized by discoloration of the skin and severe neuropathic pain, while higher concentrations can give rise to Minamata disease with auditory and visual problems, ataxia, and language difficulties [64]. Another heavy metal of great concern is lead, whose presence in the past was due to lead paints (banned since the 1970s), leaded petrol (banned since 2001), and pipes, and to companies, especially those producing batteries and welding. Poisoning from lead is defined as “saturnism” and the toxicity derives from the fact that this heavy metal binds to hemoglobin and is distributed in a multi-compartmental manner. At the level of the kidneys, liver, and spleen, lead has a half-life of one or two months, while at the bone level it replaces calcium, remaining there for a couple of decades and causing toxicity even years later. Poisonings manifest themselves with hemolytic anemia, nausea, vomiting, abdominal pain, seizures, and kidney failure [65].

### 2.11. Items of Interest for Honey Characterization

Only ten elements make up most of the Earth’s mass, with oxygen, silicon, and aluminum accounting for over 80%. Sodium and potassium, alkali metals in group 1A, are abundant in water, with sodium being the main component of seawater. Potassium is crucial for plant growth as it activates enzymes essential for protein synthesis, metabolism, and growth. Calcium and magnesium, part of group 2A, are essential for plants, with calcium aiding in cell division and magnesium being vital for chlorophyll’s structure and photosynthesis. Aluminum, abundant in the Earth’s crust, and boron, a rarer element, are also significant [66,67].

Transition metals like chromium, manganese, iron, cobalt, nickel, copper, and zinc are essential for both plants and humans. Chromium regulates glycemic processes in humans, while manganese supports enzyme function and bone and skin growth. Iron is crucial for oxygen exchange, cobalt is part of vitamin B12, and nickel is essential for hydrogenase enzymes. Copper is important for enzyme processes, neuronal myelin, and oxidative damage prevention, while zinc is necessary for plant hormone production and human growth and immunity [68,69,70].

### 2.12. Inorganic Component in Honey

Minerals, as described previously, are formed through geological processes that occur over time and are of great importance for both humans and plants, as they regulate numerous physiological processes. Based on their required quantities, minerals are classified into three categories:

- Major elements (Na, K, Ca, Mg, P, S, and Cl), required in humans in concentrations greater than 50 mg/g;
- Trace elements (Fe, I, F, Zn, Cu, Mn, Co, and Ni), required in concentrations lower than 50 mg/g;
- Ultra-trace elements (Al, As, B, Bo, Sn, and Pb) present in quantities ranging from 50 ng/g to 1 µg/g [51].

The metal content of honey is generally very low, typically ranging from 0.1% to 0.3%, though it can reach approximately 1% in honeydew honeys. This elevated metal content in honeydew honeys is attributed to their uptake through the collection of phloem sap [71]. Potassium is the most abundant metal in honey, constituting about one-third of the total mineral content, followed by sodium, while zinc, magnesium, iron, calcium, and copper have lower values [51].

The percentages of minerals and elements in honey vary depending on the plant source, geographical origin, and composition of the soil where the plants grow. The presence of non-essential heavy metals, which should be present within the product only at very low levels, can also be defined as a bioindicator of pollution of the area where honey plants grow. Polluted soil, air, and water are often the result of uncontrolled human activities, such as intensive agricultural practices. These elements are absorbed by the plants and stored within the nectar that will be used by bees [39]. Honeys produced near steel mills, roads, and cities have proven to be richer in Cd, Al, Cu, Ba, Mn, Ni, and Pb than those produced in less anthropized areas [51]. Another important factor to consider in the concentration of metals in honey is the age of the hive. The major elements, but lead as well, depend heavily on its age and, in particular, higher concentrations are found in four-year-old hives [72]. Furthermore, there is also a correlation between the presence of minerals and the antioxidant activity of honey. The antioxidant products in honey are numerous, in particular enzymes, such as catalase and glucose oxidase, ascorbic acid, phenols, substances similar to carotenoids, and various organic acids. The main positive correlation is between the content of Co, Cr, Fe, and Zn and the total phenolic concentration, while this is not present in the case of flavonoids [73]. These data are also confirmed by the shades taken on by the honeys; as already mentioned, when talking about color, darker honeys have a higher concentration of metals than the clear ones and, therefore, better antioxidant activity.

## 3. Procedures for Metal Characterization

### 3.1. Pretreatment

While reading the literature, sample preparation methodologies were found to vary depending on the type of subsequent analysis. A common initial pretreatment involves slightly heating the honey, either in water baths maintained at 40 °C [74] or through sonication. This step is crucial for reducing the viscosity of the honey, resulting in a more homogeneous product that is easier to weigh.

Attempts to freeze-dry aliquots of honey have also been described, as described for instance by Caroli et al. [75], but this process proved unsuccessful, obtaining a very compact waxy mass; the author hypothesizes that this is due to the temperature of extremely low freezing of glucose (−41 °C) and fructose (−48 °C).

Moreover, honey samples are routinely subjected to decomposition prior to the determination of metals, despite their high solubility in water. The destruction of the organic component of honey allows you to extract the inorganic matter present in it and, moreover, prevents any variation in chemical–physical parameters and interferences that could occur in the atomizer of optical or mass spectrometers, such as flame or plasma. The residues obtained from this treatment are redissolved in water or in an acidic matrix and are ready for analysis [39]. The main procedures used for matrix decomposition are described hereafter.

### 3.2. Sample Decomposition

Mineralization is a critical step in sample preparation, which involves the complete destruction of the organic matter, allowing the release into solution of the inorganic components which will be subsequently determined. Generally, sample decomposition occurs by incineration, or through an acid mineralization of the sample [39]. The main sample preparation techniques are summarized in Table 1.

### 3.3. Incineration

Methods involving incineration exploit very high temperatures (500–600 °C) to guarantee the complete degradation of the organic matter, which is transformed, generally, in carbonates and oxidized forms [76]. The phases of dry incineration are as follows: (i) drying of the sample at 100 °C; (ii) evaporation of the volatile fraction; (iii) progressive oxidation of the non-volatile fraction until its complete destruction.

This method of sample preparation requires only a few reagents, simple-to-use equipment, and minimal operator intervention [77]. In this work, the authors prepared a sample of 5 g of honey, which was placed for twelve hours in an oven at 600 °C until it became a white ash [78]. The ashes obtained were added to a mixture of nitric acid (HNO_3_) and hydrogen peroxide (H_2_O_2_) and heated to ensure the complete elimination of the organic matrix. Paramas et al., however, used a sample of 15 g of honey which was incinerated at 550 °C and then dissolved in a solution of equal parts of nitric and hydrochloric acid [79].

### 3.4. Acid Mineralization

Acid mineralization can occur according to conventional methods or using microwaves. The conventional method consists of adding a defined quantity of hot concentrated acid to the sample vessel on hotplates. In most cases, the acid used is nitric acid, but mixtures can also be used of nitric acid and perchloric, or hydrofluoric, hydrochloric, or sulfuric acid to obtain a better result. To our knowledge, in the literature, there is only one article that used acid mineralization with the conventional method [50].

Microwave digestion relies on microwaves, electromagnetic radiation with one wavelength between radio waves and infrared. The frequency of these waves ranges between 0.3 and 3 GHz. Energy can be absorbed by the sample either through electrical conduction, i.e., the migration of ions placed within an electromagnetic field, or through the rotation of the molecule dipoles. The amount of heat generated is directly proportional to the ability of the ions and dipoles to align with the applied field.

The polarization of water varies under an alternating electric field, a phenomenon that occurs simultaneously and integrally throughout the system. This process is facilitated using containers that are transparent to microwaves and therefore do not heat up. The materials most often used are polytetrafluoroethylene (PTFE) or quartz [80].

Acid mineralization is preferable, compared to ashing, for those samples that present volatile species such as Cd, Ni, Pb, and Zn [39]; also, this technique allows the use of significantly smaller quantities of sample than incineration: in fact, Di Bella et al. used 0.4 g of honey [74]; Bilandžic et al., Bontempo et al., and Drivelos et al. used 0.5 g [81,82,83]; Conti et al. 0.8 g [44]; while the highest values were achieved by Pisani et al. and Squadrone et al. who used 1 g [7,84].

Table 1 reports some mineralization procedures for honey treatment.

**Table 1:** Mineralization procedures for honey.

Sample Weight (g)	Description	References
0.5	Amounts of 4 mL of HNO_3_ and 2 mL of H_2_O_2_ are added to the sample. Microwave oven mineralization (4 min, 500 W; 5 min, 1000 W; 10 min, 1400 W) is applied. After cooling, the solution obtained is diluted to 50 mL with High Purity Water (HPW).	[81]
0.5	Amounts of 5 mL of HNO_3_, 4 mL of HPW, and 1 mL of yttrium are added to the sample. After cooling, the solution obtained is diluted to 13 mL.	[82]
0.7–0.8	Amounts of 8 mL of HNO_3_ and 2 mL of H_2_O_2_ are added to the sample. Microwave oven mineralization (20 min, 600 W) is applied. After cooling, the solution obtained is diluted to 50 mL with HPW.	[85]
0.8	Amounts of 3.5 mL of HNO_3_ and 1.5 mL of H_2_O_2_ are added to the sample. Microwave oven mineralization (15 min, 120 °C; 15 min, 120 °C; 15 min, 200 °C; 15 min, 200 °C) is applied. After cooling, the solution obtained is diluted to 15 mL with HPW.	[44]
0.4	Pretreatment at 40 °C after adding 7 mL of HNO_3_ and 1 mL of H_2_O_2_. Microwave oven mineralization (30 min, 200 °C) is applied. After cooling, the solution obtained is diluted to 25 mL with HPW.	[74]
0.5	A total of 7 mL of HNO_3_ is added to the sample. After 1 h, 2 mL of H_2_O_2_ are added. Microwave oven mineralization (20 min, 0–200 °C; 15 mL, 40 °C) is applied. After cooling, the solution obtained is diluted to 25 mL with HPW.	[83]
5	Amounts of 25 mL of HNO_3_ and 10 mL of HClO_4_ are added. The acidified sample is placed on a heating plate until completely dehydrated. After cooling, it is diluted to 50 mL with HPW.	[50]
1	Amounts of 7 mL of HNO_3_ and 1 mL of H_2_O_2_ are added to the sample. After 12 h, microwave oven mineralization (25 min, 200 °C) is applied. After cooling, the solution obtained is diluted to 50 mL with HPW.	[86]
5	The sample is incinerated at 600 °C. The ashes are added to 2 mL of HNO_3_ and 2 mL of H_2_O_2_. The mixture is stirred and then heated on a hotplate to almost complete dryness. The solution obtained is diluted to 25 mL with HPW.	[78]
15	The sample is incinerated at 550 °C. The ashes are added to HNO_3_ and HCl (1:1). The solution obtained is diluted to 100 mL with HPW.	[79]
5	The sample is incinerated. The ashes are added to 5 mL of HNO_3_ and 1 mL of H_2_O_2_. The mixture is stirred and then heated on a hotplate to almost complete dryness. The solution obtained is diluted to 10 mL with HPW.	[73]
0.5	A total of 5 mL of HNO_3_ is added to the sample. After 24 h, 2 mL of H_2_O_2_ are added. Microwave oven mineralization is applied. The solution obtained is diluted to 50 mL with HPW.	[87]
1	The sample is pretreated at 40 °C and sonicated. Amounts of 4 mL of HNO_3_ and 1 mL of H_2_O_2_ are added. Microwave oven mineralization is applied	[84]
1–1.5	Amounts of 7 mL of HNO_3_ and 1.50 mL of H_2_O_2_ are added to the sample. Microwave oven mineralization is applied. The solution obtained is diluted to 50 mL with HPW.	[7]

### 3.5. Direct Analysis

A further type of sample treatment may be adopted in cases of direct analysis: the honey, after being filtered to eliminate any wax [39], is directly introduced into the container, then it is diluted with water. The analysis, however, must be conducted within a few hours, since denaturation of the proteins contained in the sample could occur, causing flocculation and cloudiness [75]. This type of analysis is rarely conducted: it allows us to reduce the preparation time, but it can be affected by analyte losses due to incomplete digestions [39].

### 3.6. Analytical Techniques

The primary techniques employed are atomic absorption spectrometry (AAS), inductively coupled plasma optical emission spectroscopy (ICP-OES) and, more recently, inductively coupled plasma mass spectrometry (ICP-MS) [88]. Table 2 shows some applications of these analytical techniques for element determination in honey samples.

The analysis of the scientific literature conducted in this work aimed to obtain data relating to the concentrations of the various inorganic species present in honey.

The main challenges encountered were the lack of consistency in the elements analyzed and, most notably, the botanical origin of honey. It was difficult to obtain a sufficient number of studies that examined the same botanical species and the same elements. As a result, in many cases, it was necessary to rely on isolated data points representing a specific element in a particular plant, which precluded their use in statistical analyses. For instance, sage honey and honeydew honey were exclusively investigated by Bilandžic et al. and Bontempo et al., respectively [81,82]. Similar issues were also encountered regarding geographical origin. It was hard to find articles that dealt with samples collected in the same zone, and, in turn, that analyzed the same elements. To obtain as much information as possible, articles that dealt with multiple regions of a given country were merged and all regions represent that country. This is the case, for example, with Paramas et al., who analyzed honeys from six different Spanish regions (Arribes del Duero, Sierra de France, Sierra de Gata, La Vera, Plasencia, and Sanabria), and whose data have all been brought together under one exclusively Spanish sample [79].

### 3.7. Analysis of Botanical Provenance

The analyzed data were evaluated without considering the geographical origin of the honey, but by examining only its botanical origin, i.e., the genus of the plant from which it is derived. The results demonstrated that the main metals present in honey are potassium (K), calcium (Ca), and sodium (Na). K reaches maximum concentrations of 5300 mg/kg in chestnut honey, while the lowest values are found in the rhododendron honey, which contains 659 mg/kg. The data, however, would need to be further analyzed, since they are based on a single value, that of Bontempo et al. [82]. Potassium is the most representative element in honey and chestnut alone accounts for almost 88% of the mineral component, as also confirmed by the authors, who claim it to be within a range of 70 to 90% [82]. A similar discussion can be made for Ca, the second most abundant element; in fact, chestnut has a range of values between 23 mg/kg and 487 mg/kg, while in rhododendron it does not exceed 17 mg/kg. It also has rather high values in acacia and in eucalyptus honey, where it reaches maximum concentrations, respectively, of 349 mg/kg and 372 mg/kg. Na has maximum average values of around 30 mg/kg (acacia, chestnut, lime, sage, and wildflower), while it has high concentrations in eucalyptus honey, reaching 112 mg/kg. This high value could be due to the fact that the samples analyzed were Italian [74,82] and, in the case of Di Bella, come from Sicily and Calabria; the marine spray may have influenced the soil of the area and, consequently, also the supply of Na in the plant. Other elements are also generally always detectable. Mg is particularly abundant in honeydew, where it reaches a peak of 79.0 mg/kg, followed by chestnut honey (59.1 mg/kg), wildflower (40 mg/kg), and lime (25.5 mg/kg). The lowest values are found in acacia and citrus honey. Zn and Fe are the last two elements particularly abundantly present in honey. The highest values of Zn are found in eucalyptus honey, with a range between 0.8 mg/kg and 9.51 mg/kg, and in lime (6.78 mg/kg) and wildflower (6.82 mg/kg), while the lowest concentrations are again found in the rhododendron honey with values around 1 mg/kg. Fe, on the other hand, has slightly lower values than Zn, but again the highest concentrations are present in chestnut honey (0.602–7.16 mg/kg) and the lowest ones in rhododendron honey (0.57–1.1 mg/kg). In other botanical species the average Fe value is around 3.50 mg/kg. In this analysis, the Fe and Zn values of Perna et al. were not taken into consideration [73], since they went far beyond the values collected by the other authors. According to the authors, these very high values, for example 27.3 mg/kg of Fe or 17.9 mg/kg of Zn in chestnut honey, compared to the maximum concentrations obtained from the literature of 7.16 mg/kg and 3.5 mg/kg, respectively, are to be attributed to contamination due to beekeepers’ work tools and to the environment. Oroian et al. add that the higher Zn concentration could also be due to honey collection and its storage inside galvanized containers [78]. Despite being a non-essential element, aluminum (Al) is present in high concentrations, due to its presence in abundant quantities at an environmental level [7]. The highest values are found in chestnut (2.25–11.5 mg/kg), and in citrus fruit, eucalyptus and wildflower honey, while the lowest values are achieved by acacia honey (0.60 mg/kg).

Chromium (Cr), lead (Pb), and nickel (Ni) are elements present as contaminants within honey. High levels of these elements are due to the presence of anthropogenic environmental pollution, such as machine exhaust or exhaust fumes released by steel mills, as also hypothesized by Perna et al. and Solayman et al. [51,73]. The elements are absorbed from the soil through the plant roots and are later found in pollen and consequently in honey. Mn can be considered an element linked to the natural presence of oxides in the soil, but it is also linked to environmental pollution; Mn can be released by industrial fumes, deriving from fossil fuels or gasoline additives such as methylcyclopentadienyl manganese tricarbonyl (MMT) [78]. Pb reaches the highest values in chestnut, with a range between 0.01 mg/kg and 0.49 mg/kg, while the lowest concentrations are present in acacia honey, with a maximum of 0.062 mg/kg.

With Regulation 1881/2006 of the European Commission, maximum allowable levels of Pb were introduced in some foods; however, honey did not appear [89]. The levels were updated in 2015 with Regulation 2015/1005, due to the need to regulate the high but irregular Pb contents present in many foods, including honey [90]. A maximum limit of 0.10 mg/kg was set. According to this limit, the values found in the literature would be illegal, but these data are prior to the second regulation, i.e., they date back to a period in which the maximum Pb limit was not yet set. The data in question were found by different authors, in particular, in sage honey, but also in almost all other types of honey (chestnut, wildflower, citrus, eucalyptus, and molasses). More recent data, again Italian, find maximum values of 0.071 mg/kg in acacia honey, a value perfectly in line with current legislation [7]. The highest levels of Cr are found in acacia honey (<LOD 0.94 mg/kg) and the lowest ones in lime tree honey, where it reaches the maximum value of 0.067 mg/kg, as determined by Squadrone et al. Contamination could also take place during honey harvesting, since, due to its acidity, it could corrode the stainless steel of the tools, releasing Cr into the honey itself [78].

Ni is found at rather low concentrations at an environmental level, despite being widely distributed, and could result from anthropogenic actions such as the use of fertilizers for soil or low-quality plant foliage [78]. The highest levels of Ni are present in lime honey (0.04–0.325 mg/kg), while the lowest ones are in rhododendron honey (0.1–0.15 mg/kg). Also interesting is the analysis of the alkaline metal rubidium, which, as observed by Squadrone et al. [10], is seldom analyzed at a bromatological level, but widely distributed in the Earth’s crust, reaching an abundance like that of Zn. Its concentrations vary depending on the soil in which the plant grows; therefore, it could be a good indicator of the origin of honey. Unfortunately, this metal has only been analyzed by two authors [7,82], who agree that the highest levels are found in chestnut honey, while the lower ones are in the acacia.

**Figure 2:**
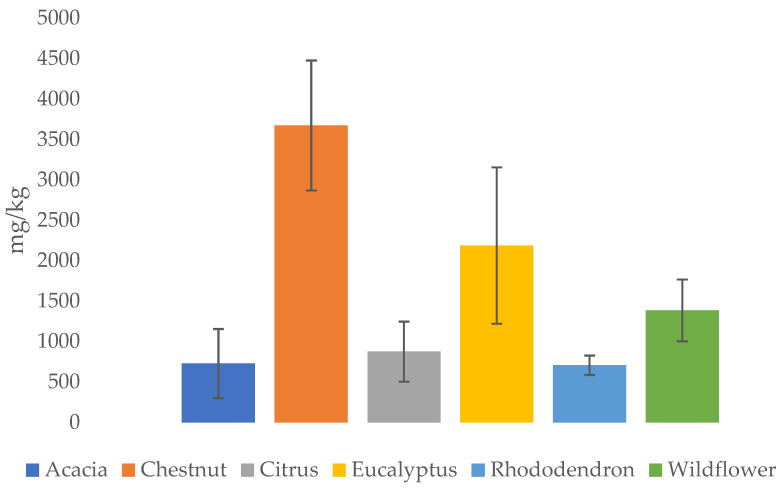
Average metal content (mg/kg) in honey of different botanical origin (all figures presented in this work were created by the authors based on the data analyzed).

As summarized in Figure 2, honey containing the highest content of elements is chestnut honey, followed by eucalyptus, wildflower, citrus, acacia and, finally, rhododendron honey. This would also confirm that honeys containing more metals are also those with darker colors.

They seem to be particularly related to the high presence of metals such as Ca, Cd, Fe, and Pb [91]. Dark and amber honeys such as chestnut, in fact, contain higher concentrations of metals, while rhododendron honey, which takes on a color from white to straw yellow, it is actually the poorest in inorganic material. The few data obtained through the literature did not allow us to delve deeper into the discussion of honeydew, which, given its production, should be one of the richest in metals, like chestnut honey. Table 3 summarizes the ranges of element concentrations in honeys from different botanic origins.

### 3.8. Investigation of Geographical Origin

Further data analysis was carried out by evaluating only the geographical origin of the honey, grouping honeys of different botanical origins under the same state. The three most expressed elements are confirmed to be K, Na, and Ca. It is interesting to analyze the case of Egypt first: the honey coming from this country has the highest concentration of elements compared to any other country, with values that are double the maximum values found in all other honeys. For example, in Egypt, K reaches maximum values of 15,550 mg/kg, while Turkey, which is the country with the second-highest value, reaches 7030 mg/kg. Analyzing the article by Rashed et al., from which these values have been extrapolated, it can be noted that they also found, among the honeys analyzed, some from hives where bees were fed with syrup [93].

The authors justify these values by saying that they are due precisely to how the bees were fed, since the syrup contains inorganic salts of Cl, K, Mg, Na, and Mn. Furthermore, they state that high concentrations of sugars lead to an increase in the concentration of K, Fe, and Zn. Contamination by Fe, Pb, and cadmium (Cd), however, could have occurred during technological processes in the syrup production, due to the addition of calcium hydroxide (Ca(OH)_2_), calcium phosphate (Ca_3_(PO_4_)_2_) and sulfur dioxide (SO_2_), which could partially corrode the containers, dissolving them in the sugary mixture. It is important, however, to also consider the Egyptian “environmental” situation of that period.

In the article “Environmental health in Egypt” by Wagida Anwar [94], published in 2003, i.e., a year before the honey analyses, Egypt is described as in the midst of the ecological problems of developing countries. The average concentrations of Pb in the air fluctuated, in the 1990s, between 4 µg/m^3^ (1994) and 12 µg/m^3^ (1997) in the center of Cairo and hematological analyses of traffic policemen demonstrated Pb values that exceeded 60 µg/100 mL of blood. These values in the air dropped, in 2001, below 1 µg/m^3^ thanks to health interventions, the monitoring of road activities, and the use of unleaded petrol. The accumulation in the ground, however, could justify an average value of 4.06 mg/kg in honey, with a maximum of 8.03 mg/kg.

Completely different values are present in Saudi Arabia. Metal concentrations are significantly lower than the Egyptian ones and, in some cases, even relatively low compared to other states in the world. In fact, compared to all the data, Saudi Arabia honeys present the lowest concentrations of K and Ca. Such low concentrations of elements could be due to the type of honey considered, in particular, the wildflower honey analyzed by Osman et al. [95], and the acacia, lavender, and ziziphus honeys analyzed by Al-Khalifa et al. [96]. All these honeys have very light colors and, therefore, a lower concentration of inorganic material.

Turkey has a medium-high level of heavy metals. The average concentration of Cd is 0.145 mg/kg, exceeded only by India (0.300 mg/kg) and New Zealand (0.230 mg/kg); that of Cr is 0.496 mg/kg, the second-highest value after Spain; and Pb concentrations reach 0.327 mg/kg, only exceeded again by India (0.485 mg/kg), and, as already addressed previously, by Egypt. Also, Turkey presents one of the highest values of Ni, 1.24 mg/kg, surpassed only by Egypt (1.34 mg/kg) and Sudan (2.03 mg/kg). Similarly to what was said for Egypt, Turkey and India also present significant environmental pollution that could have a massive impact on air, water, and soil, and consequently the inorganic matter of honey.

Data relating to honey produced in China demonstrate a similar concentration of metals to those found in European states, except for Na, which has particularly low concentrations (9.52 mg/kg). The heavy metals analyzed are present only in trace amounts. These data could be due to the place of collection of the honey samples; in fact, the only article that covered the elements of interest uses an ICP-MS-based ionomics method for discriminating the geographical origin of honey of *Apis cerana Fabricius*, in 2021 by Wu et al. [87], where the samples of honey were collected in three areas of the Qin Mountains (Liuba, Yangxian, and Longxian), in northern China, not particularly polluted places.

The Balkans bring together honeys from Slovenia, Greece, and North Macedonia. In the literature, there are also, for these states, values regarding Al, an element that is poorly determined in honey but is abundantly present in the soil; its average value is 1.20 mg/kg, similar to the Bulgarian one (0.91 mg/kg) and much lower than the Italian level (2.35 mg/kg). Values again like the Italian ones are found for K, Na, and Cu, while they are clearly different from the Bulgarian ones. K in Balkan honey reaches average values of 2328 mg/kg, compared to 866 mg/kg for the Bulgarian one; Na (77.9 mg/kg) and Cu (1.28 mg/kg) have concentrations, respectively, seven times and almost forty-six times higher than those present in Bulgarian honey (11.8 mg/kg for Na; 0.028 mg/kg for Cu)

Concentrations being so similar to those found in Italy could be due to the shared soil, with the two being relatively neighboring nations, bordering as far as Slovenia is concerned. The high levels of Mn (13.7 mg/kg) and Pb (1 mg/kg) could instead be due to human pollution.

The situation of the metal levels present in Spain is also interesting. Honey from that country presents some of the highest concentrations found in the literature. As shown in Table 4, it has the highest concentrations of Cr, Mg, and Na. Cr has an average value of 1.11 mg/kg, reaching a peak of 4.48 mg/kg. The concentration of Mg is second only to Egypt and ranges between 3.60 and 1079 mg/kg, while Na has an average value of 202 mg/kg, which is double the levels present in the other states analyzed. Elements such as Mn, Fe, and Cu also have rather high values, if compared to other European states. Finally, Spain is the only country in which it is possible to determine Cl within honey and this could be a useful element in geographical discrimination.

Italian honey has a rather high total concentration of minerals, just below the Turkish, Spanish, and Balkan ones. The most present element is K, with a range between 147 mg/kg and 4136 mg/kg, followed by Ca, which has the highest average values compared to all other countries, 224 mg/kg. The quantities of Mg, Cu, and Fe are also higher compared to other states; for example, in Italy Mg is on average 80.9 mg/kg, almost double that of Turkey or Poland, but again similar to the Balkan concentration (93.2 mg/kg). As already highlighted in the botanical analysis, the value of Pb again exceeds the maximum limits of Regulation 2015/1005 (0.1 mg/kg) in different samples.

The other heavy metals such as nickel, chromium, and cadmium, however, are in line with the values of most of the other states analyzed.

**Figure 3:**
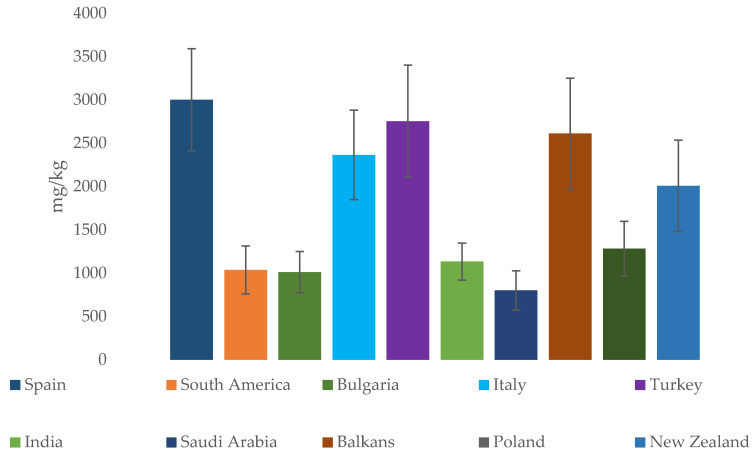
Average metal content in honey from different countries and regions (all figures presented in this work were created by the authors based on the data analyzed).

As demonstrated in Table 4 and summarized in Figure 3, the states that have the highest concentrations of metals in honey are Turkey, for the Asian area, and Spain, the Balkan area, and Italy as far as Europe is concerned. However, as also stated by Pisani et al. [84], it is complicated to make good comparisons between data in the literature, since there are many variables that depend on both the different honeys that can be used—which, as reported in the botanical analysis, have different concentrations of metals—and on the sample preparation techniques, which could influence the values obtained.

## 4. Chemometric Treatment

### 4.1. Analysis of Botanical Provenance

A chemometric analysis of the concentrations found in the literature was performed by Principal Component Analysis (PCA) with XLStat 2007.3 software, as a Microsoft Excel add-on. All the data were normalized before being statistically processed to ensure consistency and comparability across the various studies.

**Figure 4:**
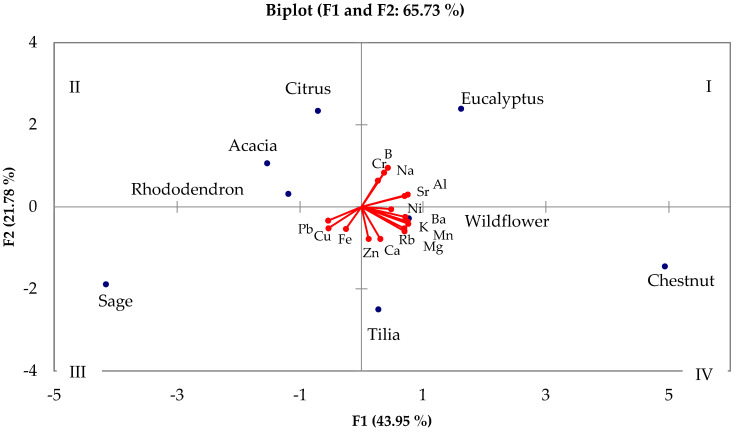
Combined plot of scores and loadings obtained by PCA considering the botanical origin of honey samples. (All figures presented in this work were created by the authors based on the data analyzed).

Figure 4 reports the combined graph of scores and loadings obtained from the metal content reported in the literature, considering the samples with different botanical origins. The figure shows that the results obtained are distributed based on two main components, F1 and F2. From the distribution of loadings, it can be hypothesized that these may be linked, respectively, to terrigenous and anthropogenic elements. The two main components alone explain 65.73% of the total variance. From Pearson’s coefficients, a high correlation is observed among Al, Ba, K, Mg, Mn, Rb, and Sr: these elements, which develop along the F1 axis, are those that are mainly influenced by the characteristics of the soil in which the plant grows. The F1 component alone represents 43.95% of the total variance and includes most variables with a positive correlation, except for Pb, Cu, and Fe which show negative correlation. This behavior could be due to variations in pollutants in the area in which the honey was produced (Pb) and to pesticides used (Cu and Fe).

The F2 component explains 21.78% of the total variance. Na is the only element that presents a positive correlation with this factor. The concentration of sodium is very much conditioned by the proximity to the sea, so its greater concentration in some honeys is explained by the influence of sea spray.

As far as honey is concerned, it can be noted that chestnut, honeydew, linden, and millefiori honeys are the ones richest in metals, demonstrating their high nutritional power as sources of minerals. This confirms why honeydew is considered particularly rich in mineral salts and trace elements and therefore recommended as a restorative supplement, as well as a sweetener. Sage honey samples contain the highest amounts of Cu, Fe, and Pb, confirming data reported in the literature: sage is a botanical species that could be strongly affected by an environment rich in these elements.

Chestnut is confirmed as being the honey richest in metals, followed by honeydew, and it develops mainly along the F1, while rhododendron, citrus, and acacia, on the other hand, are the poorest in metals. It can also be noted that Na has a correlation with eucalyptus honey, probably due to its cultivation in marine areas that influence the growth soil.

Particularly interesting, however, is the characterization of sage, the only botanical species which develops on F2, i.e., the component associated with pollutants. Sage honey, unlike the others, is the one most correlated to the presence of Pb and Cu and this is a significant and distinctive aspect. It is capable of accumulating large quantities of heavy metals and pollutants within its tissues and is therefore used as a bioindicator for biomonitoring studies, together with other traditional medicinal plants, such as, for example, St. John’s wort, mallow, calendula, garlic, and mint. In particular, Angelova et al. categorize sage as a plant that hyperaccumulates Pb and accumulates Cd and Zn, therefore confirming the obtained results [97].

The same dataset was also processed using Hierarchical Ascending Classification (HAC). This allows us to divide a dataset into subsets, where the units belonging to the same subset are as homogeneous as possible with each other.

**Figure 5:**
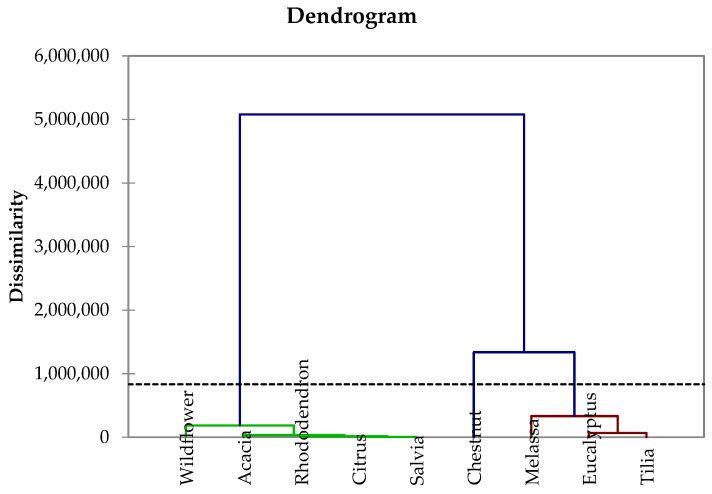
Dendrogram obtained by HAC considering the botanical origin of honey samples (all figures presented in this work were created by the authors based on the data analyzed).

This analysis, as demonstrated in Figure 5, highlights an interesting detail: in fact, the dendrogram obtained divides the honey samples into two main clusters, composed in one case of arboreal plants and in the other of herbaceous plants, shrubs, and small trees. Acacia is an exception: although it is a large tree, it falls into the second cluster. This behavior derives from the fact that *Robinia pseudoacacia L*. belongs to the *Fabaceae* family and is considered a pioneer plant, that is, capable of colonizing even nutrient-poor soils; this could explain why its honey is among the poorest in elements.

### 4.2. Analysis of Geographical Provenance

PCA was carried out considering all the datasets referring to the geographical origin; the first two components were used to describe the entire dataset and alone represent 62.91% of the total variance. F1 accounts for 46.30% of the variance.

**Figure 6:**
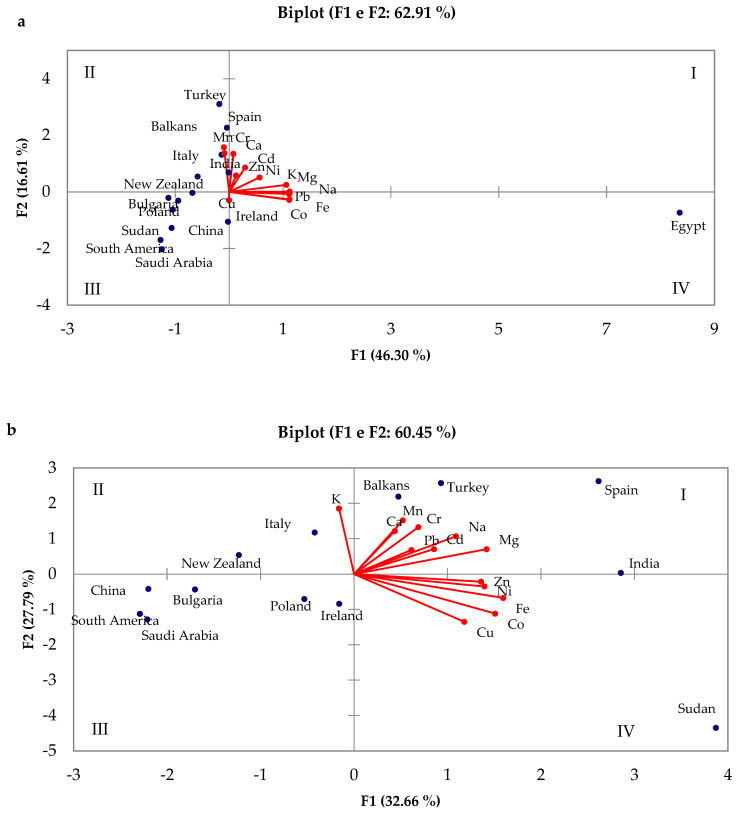
Combined plot of scores and loadings obtained by PCA considering the botanical origin of honey samples (**a**) with and (**b**) without the honey sample from Egypt (all figures presented in this work were created by the authors based on the data analyzed).

As shown in Figure 6a, the distribution of the scores is strongly influenced by the high metal content of honey from Egypt due to, as explained before, the glucose syrup adopted to feed the bees. In the presence of the Egyptian data, the chemometric analysis would be very complex and staggered, preventing a correct interpretation of the results.

So, the chemometric analysis was also conducted without considering the data from Egypt (Figure 6b). In this case, the first two components represent 60.42% of the total variance and F1 accounts for 27.79% of the variance. All the elements are positively correlated with F1, with the only exception of K.

In this case, a good correlation can be observed among the transition elements Co, Cu, Fe, Ni, and Zn.

The countries are not easily distinguishable from each other; this could be because the botanical origin of the honeys analyzed was not considered, and data from samples deriving from different plants but from the same country were averaged.

It is only possible to see that, from the first to the fourth quadrant, different groups can be observed: (i) Balkans, Turkey, Spain, and India (on the F1 component); (ii) Italy and New Zealand; (iii) China, Bulgaria, Poland, Ireland, South America, and Saudi Arabia located in the opposite direction in comparison with the loadings; and (iv) Sudan alone in the direction of the transition metals.

In the future, it would be interesting to evaluate how these correlations would change if honeys of the same botanical origin but from different states were analyzed.

This results dataset was also treated with Hierarchical Ascending Classification, but no significant information was obtained, so the graph was omitted. (All figures presented in this work were created by the authors based on the data analyzed).

## 5. Conclusions

In the literature, it is well known that some components present in honey lead to information about its origin, for example aliphatic organic acids, amino acids, aroma compounds, aromatic carbonyl compounds, oligosaccharides, phenolic acids, and esters, specific isotopic ratios (e.g., D/H) for botanical origin. For geographical origin, amino acids, aroma compounds, flavonoids, oligosaccharides, and specific stable isotopic ratios (e.g., 18O/16O) could be very useful. Less has been written regarding the content of inorganic elements. There are numerous studies on the characterization of honey from the point of view of the content of macro and micro elements, but few have the aim of evaluating the use of these parameters for provenance and authentication studies. From our investigation, it emerged that the content of inorganic elements was less effective in discriminating geographical origin. Currently, all analysis systems, including pollen analysis, have limitations in identifying the botanical origin. In the case of the latter, the wording of the directive is far from complete and leaves ample room for interpretation. It is in fact stated that an indication relating to floral or vegetal origin can be applied if the product comes mainly from that origin and possesses its organoleptic, physical–chemical, and microscopic characteristics. These are reported in the scientific and technical literature but, for the moment, have not been introduced into legislation. The ideal system would consist of identifying, for each possible botanical origin, one or more “markers”, that is, substances that are present exclusively in the nectar of the species in question, in constant quantities and not modifiable by the processes of processing by bees, extraction, and conservation. Metal content has proven suitable for gaining insight into the botanical origin because the elements are strongly influenced by the bioaccumulation capacity of the individual types of plants from which the nectar is taken. The combination of this parameter with other components, such as the composition of the soils in the area where plants harvested by bees grow, especially with the support of modern statistical data evaluation techniques, seems to be a promising approach for proof of honey’s authenticity.

## Figures and Tables

**Table 2:** Analytical techniques for inorganic element quantification.

Method	Elements	References
FAAS	Ca, Fe, K, Mg, Na, Zn	[81]
FAAS	Ca, Fe, K, Mg, Na	[85]
GFAAS	As, Cu, Cd, Pb, Se	[81]
GFAAS	Cu, Mn	[85]
ICP-MS	As, Cd, Cr, Cu, Fe, Mn, Ni, Pb, Zn	[75]
ICP-MS	As, Be, Ca, Cd, Co, Cr, Cu, Fe, K, Mg, Mn, Na, Ni, Pb, Se, Tl, U, V, Zn	[44]
ICP-MS	As, Cd, Cr, Cu, Mn, Ni, Pb, Se	[74]
ICP-MS	Ba, Ce, Co, Cu, Dy, Er, Eu, Gd, Ho, La, Li, Lu, Mg, Mn, Na, Ni, Pb, Pr, Sm, Sr, Tb, Y, Yb	[83]
ICP-MS	As, Bi, Cd, Ce, Dy, Er, Eu, Gd, Ho, In, La, Lu, Nd, Pb, Pr, Sc, Se, Sm, Tm, Y, Yb	[86]
ICP-MS	As, Cd, Cr, Cu, Hg, Fe, Mn, Ni, Pb, Zn	[78]
ICP-MS	As, Ba, Cd, Co, Cu, Ni, Pb, Sb, Th, U	[84]
ICP-MS	Ag, Al, As, Be, Bi, Ce, Cd, Co, Cr, Cu, Dy, Er, Eu, Fe, Ga, Gd, Ho, In, La, Lu, Mn, Mo, Nd, Ni, Pb, Rb, Sb, Sc, Sm, Sn, Tb, Tl, Tm, U, V, Y, Yb, Zn	[10]
ICP-MS	Al, B, Ca, Cd, Co, Cr, Cu, Fe, K, Mg, Mn, Mo, Na, Ni, Pb, Ti, V, Zn	[87]
ICP-OES	Al, B, Ba, Ca, Cr, Cr, Cu, Fe, K, Mg, Mn, Na, Ni, Pb, Rb, Sr, Zn	[82]
ICP-OES	Cu, Fe, Mn, Zn	[75]
ICP-OES	Al, Ca, Fe, K, Mg, Ni, Pb, Se	[74]
ICP-OES	Cu, Fe, P, Pb, S, Zn	[50]
ICP-OES	As, Cd, Co, Cr, Fe, Mo, Pb, Zn	[73]
ICP-OES	Ca, Fe, K, Mg, Mn, Na, Sr, Zn	[84]

**Table 3:** Concentration ranges (minimum and maximum value, if available) of elements in different honey types, expressed in mg/kg.

Honey Type	Al	B	Ba	Ca	Cr	Cu	Fe	K	Mg	Mn	Na	Ni	Pb	Rb	Sr	Zn	References
**Acacia**	0.52 0.60	3.70	<LoD	15 349	<LoD 0.94	0.12 0.30	0.50 2.81	305 1150	5 8	0.20 1.71	4.1 33.9	0.19	<LoD 0.06	0.8 1	n.d.	0.21 2.42	[10,74,78,81,82,92]
**Chestnut**	2.25 11.5	4.50	0.70	23 487	<LoD 0.83	0.07 0.81	0.60 7.16	2824 5300	49 59	1.50 8.30	11.9 35.8	0.04 0.21	0.01 0.49	23 43	0.50	0.66 5.11	[7,73,74,78,81,82,92]
**Citrus**	0.70 8.50	6.20	<LoD	36 289	<LoD 0.76	0.06 0.20	0.90 3.94	186 3110	6.0	0.2 0.81	17.1	0.08 0.1	<LoD 0.37	1.0	0.20	0.5 3.94	[73,74,82]
**Eucalyptus**	2.0 9.28	6.40	0.20	112 372	<LoD 0.53	0.17 0.98	1.67 3.53	1240 4940	18	1.65 1.80	112.4	0.1 0.21	<LoD 0.11	2.0	0.4	0.8 9.51	[73,74,82]
**Molasses**	3.80	5.80	0.10	51.0	<LoD	1.20	1.50	2375	79.0	2.30	11.8	0.20	0.10	11.0	0.10	1.50	[82]
**Linden**	1.80	n.d.	n.d.	388	0.03 0.07	0.34 0.76	0.65 4.02	1574	25.5	1.29 3.20	31.9	0.04 0.32	0.01 0.08	8.6	n.d.	1.0 6.78	[74,78,81,82]
**Rhododendron**	1.20 2.10	2.00	0.10	17.0	<LoD 0.09	0.20 0.96	0.57 1.10	659	15.0	1.40 2.20	3.70	0.1 0.15	0.02 0.10	4.2 5	<LoD	0.97 1	[10,82,92]
**Sage**	n.d.	n.d.	n.d.	173.9	<LoD	<LoD	4.17	769	11.6	n.d.	31.80	n.d.	0.51	n.d.	n.d.	0.94	[81]
**Wildflavour**	1.80 7.86	5.00	0.20	59 387	<LoD 0.52	0.18 0.95	1.54 3.63	270 2460	27 40	0.55 2.30	10.2 36.1	0.10 0.22	0.03 0.17	5.9 8	0.10	1.02 6.82	[10,73,74,78,81,82]

n.d. = not detected.

**Table 4:** Concentration ranges (minimum and maximum value, if available) of elements in honey by geographical origin, expressed in mg/kg. Element concentration (mg/kg).

Country/Region	Al	Ca	Cd	Co	Cr	Cu	Fe	K	Mg	Mn	Na	Ni	Pb	Zn	References
**Ireland**	n.d.	111 125	n.d.	n.d.	n.d.	1.65	8.00 19.0	562 566	31 36.1	4 5.55	98 119	n.d.	n.d.	5 12.5	[39,84]
**Spain**	n.d.	13.8 341	0.001 0.36	0.001 0.72	0.001 4.48	0.04 7.80	0.84 60	14 6785	3.60 1079	0.01 27	11 1221	0.01 3.37	<LOD 1.20	0.07 19.1	[39,51,79,84]
**South America**	n.d.	6.92 77.4	0 0.10	n.d	n.d	0.18 0.38	1.4 5.7	832 1023	17.3 26.3	0.37 2.61	32.2 48.0	<LOD 0.40	<LOD 0.20	0.85 2.00	[10,44,51]
**China**	n.d.	78.2	0.017 0.021	0.005	0.01	0.17 0.44	2.88	1673	45.1	1.81	9.52	0.06	0.03 0.05	1.27 1.40	[51,87]
**Italy**	0.52 2.6	9.10 409	0.001 0.30	0.002 0.057	0.01 0.10	0.14 5.90	0.30 35.1	147 4136	3.90 159	0.08 16.9	6.10 232	0.04 0.17	0.003 1.533	0.18 8.12	[10,39,51,74,84]
**Turkey**	0.004 28.7	3.30 900	0 0.34	0.001 0.03	0.001 1.04	<LOD 3.50	0.04 19.7	143 7030	2 111	<LOD 74.2	9.30 172	<LOD 2.17	<LOD 1.20	<LOD 20.2	[39,51,84]
**India**	n.d.	32.6 84.6	0.05 0.55	0.25	n.d	1.06 2.90	3.60 28.4	490 932	18.5 205	0.9 10.2	97.9 304	0.05 0.65	0.05 0.92	1.10 29	[39,51]
**Saudi Arabia**	n.d.	1.5 27.7	0 0.04	n.d	n.d	0.21 0.6	0.31 8.39	9.3 1367	18.4 23.2	0.03 0.37	10 133	n.d.	0.03 0.24	0.2 3	[39,51]
**Balkans**	1.2	4.10 170	0.002 0.222	0.01 0.09	0 0.4	<LOD 5.90	0.03 7	169 3323	4.40 182	0.11 82	5.90 150	0.02 1.3	<LOD 2	0.31 15	[10,39]
**Poland**	n.d.	3.3 159	0 0.03	0 0.3	0 0.5	<LOD 1.82	<LOD 16.1	7.7 3659	1.10 145	0.10 8	0.38 131	<LOD 0.5	<LOD 0.07	<LOD 22.3	[39,51]
**Egypt**	n.d.	n.d	0 0.5	1.75 3.2	n.d.	<LOD 1.75	21.5 3690	213 15,550	102 1325	0.46 5.7	378 2550	<LOD 4.10	0.86 9.30	1.63 9.3	[39,51]
**New Zealand**	0.21 21.3	7.21 94.3	0.01 0.45	n.d	0.12 0.55	0.09 0.70	0.67 3.39	34.8 3640	7.52 86.3	0.18 4.75	1.10 110	0.02 0.65	0.01 0.04	0.20 2.46	[51]
**Bulgaria**	0.24 1.58	32 110	0.01	0.01	0.01 0.02	0.01 0.04	0.35 4.37	105 1628	6.00 97	0.06 12.7	7.22 16.3	0.01 1	0.08 0.31	0.08 1.17	[51]
**Sudan**	n.d.	35.6 82.9	0.01	0.005 1.17	0.01	2.94 58.1	2.05 33.65	17.6 74.7	23.7 177	0.12 1.02	14.1 28.2	<LOD 4.06	<LOD 0.45	4.86 9.61	[51]

n.d. = not detected.

## Data Availability

Data, associated metadata, and calculation tools are available from the corresponding author (paolo.inaudi@unito.it).
